# Takotsubo cardiomyopathy in pregnancy: a case report and literature review

**DOI:** 10.1186/s12884-019-2233-7

**Published:** 2019-03-12

**Authors:** Felix Mwembi Oindi, Evan Sequeira, Herman Ryan Sequeira, Steve Kyende Mutiso

**Affiliations:** 1 Department of Obstetrics and Gynecology, Aga Khan University, P.O. Box 30270-00100, Nairobi, Kenya; 2 Department of Pulmonary and Critical Care Medicine, Norwalk Hospital/Yale University, Norwalk, CT USA

**Keywords:** Takotsubo cardiomyopathy, Preeclampsia, Pregnancy

## Abstract

**Background:**

Takotsubo cardiomyopathy is rare in pregnancy and is characterized by left ventricular dysfunction with apical ballooning. This transient cardiac dysfunction may affect women of childbearing age in the antepartum, intrapartum or postpartum period. Most patients respond well to medical management with resolution of cardiac dysfunction within weeks.

**Case presentation:**

A 35-year-old female in her second pregnancy presented with severe preeclampsia at 31 weeks of gestation. She subsequently developed severe substernal chest pain and workup showed a stress induced cardiomyopathy prior to her delivery via caesarean section. She had full recovery of her cardiac function by 12 weeks postpartum after medical management.

**Conclusions:**

Stress induced cardiomyopathy, though rare, should be considered after acute myocardial infarction has been ruled out in gravid females presenting with acute chest pain. Management should involve a multidisciplinary team. Cardiac function recovery is common within 4 weeks although some patients may require long term heart failure management.

## Background

Takotsubo cardiomyopathy (TCM) also known as stress induced cardiomyopathy is characterized by new onset left ventricular dysfunction with variable wall motion abnormalities in the absence of significant coronary artery disease [1–3]. Cardiac function usually recovers spontaneously in most patients within days or weeks [4, 5]. The common triggers of TCM include emotional or psychological stressors with a higher incidence seen in patients with preexisting psychiatric conditions [6]. However, up to 20% of affected patients have no identifiable stressors [1, 7]. Majority of TCM cases in pregnancy are described in the peripartum period irrespective of the mode of delivery [8–10] making it difficult to differentiate TCM from peripartum cardiomyopathy [5].

In this article, we present a case of a 35-year-old female who presented with severe preeclampsia at 31 weeks of gestation and subsequently developed TCM during her hospitalization. We further review the current literature on stress-induced cardiomyopathy in pregnancy.

## Case report

A 35-year-old Indian female, para 1 + 0 gravida 2, was admitted with severe preeclampsia at 31 weeks of gestation after complaints of anasarca for 1 week. She reported normal fetal movements with no epigastric discomfort, headaches or visual blurring. She had a blood pressure of 190/110 mmHg with a regular pulse rate at 70 beats/minute palpable on all extremities. She was afebrile at 36.7°C, had a respiratory rate of 18 breaths per minute and peripheral oxygen saturation of 99% on room air.

Six years prior, she had had severe preeclampsia and a preterm delivery by caesarean section at 29 weeks of gestation. The baby died of infant respiratory distress syndrome shortly after birth causing the patient significant psychological distress requiring counselling. This made her defer the possibility of a pregnancy for years sighting psychological trauma.

This pregnancy, she presented in the first trimester for antenatal care. Her blood pressure was 110/70 mmHg and antenatal profile was unremarkable: Hemoglobin 13.0 g/dl; Hepatitis-B surface Antigen, H.I.V. and Venereal Disease Research Laboratory (VDRL) were negative. Her blood group was O with positive Rhesus factor. The dating scan confirmed a 12-week-old gestation and she was placed on aspirin 75 mg throughout her pregnancy. Subsequent antenatal visits at 16, 20, 24, 28 and 30 weeks were uneventful with normal blood pressures. An anomaly obstetric scan at 20 weeks gestation showed no fetal anomalies with subsequent growth scans at 24 and 28 weeks showing normal growth with normal umbilical and middle cerebral artery doppler flows.

On admission, she had macro albuminuria with elevated urine albumin creatinine ratio of 422 mg/mmol. Her hemoglobin was 12.1 g/dl and the platelet count was 194,000/ml. The renal and liver functions were normal.

She was started on intravenous (IV) magnesium sulphate infusion as per the Zuspan regime [11] (4 g slow IV bolus followed by 1 g/hour for 24 h) for maternal seizure prophylaxis and fetal neuro-protection [12]. She received multiple push doses of IV hydralazine for blood pressure control and was eventually placed on hydralazine infusion at 1 mg/hour. In addition, she received oral nifedipine and oral alpha methyldopa. Two doses of betamethasone 12 mg were administered intramuscularly 24 h apart for fetal lung maturation. She also received low molecular weight heparin (LMWH) for thromboprophylaxis and the total fluid intake was maintained at 80 ml/hr. to minimize the risk of pulmonary oedema.

**Fig. 1:**
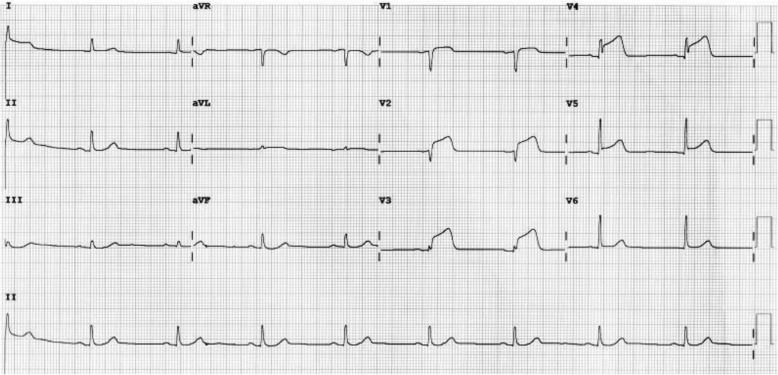
ECG trace showing ST elevation in the antero-septal leads (V1–4), Q waves in the septal leads (V1–2) and peaked T waves in V2–4) indicating an acute antero-septal ST-Elevation Myocardial Infarction

**Fig. 2:**
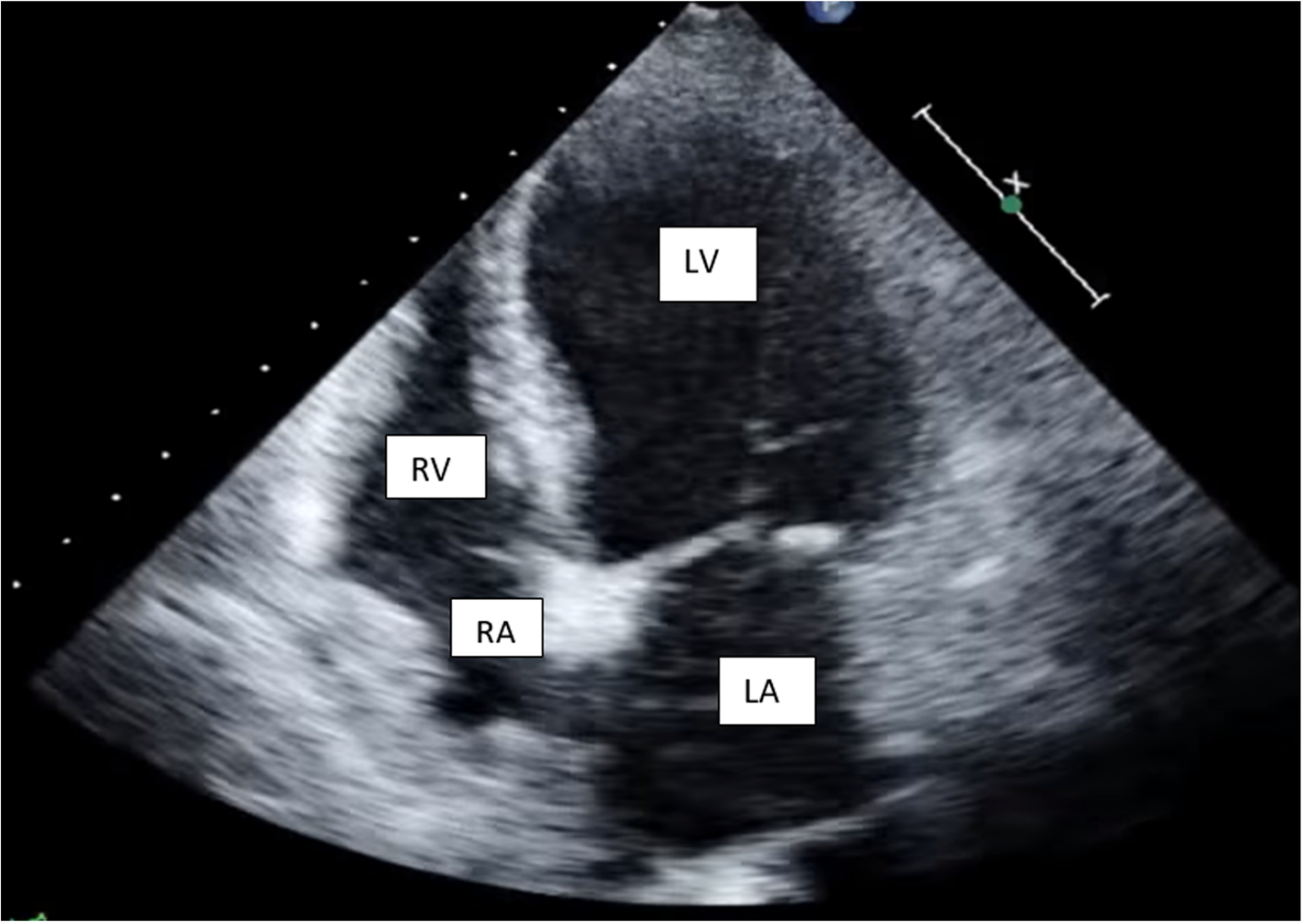
Transthoracic echocardiography showing apical ballooning of the left ventricle (LV: Left Ventricle; RV: Right Ventricle; LA: Left Atrium; RA: Right Atrium)

Thirty hours post-admission, she developed worsening orthopnea with severe sub sternal left sided chest pain radiating to the left axilla without symptoms of diaphoresis, nausea or vomiting. Her blood pressure was 138/82 mmHg, pulse rate of 64 beats per minute, and peripheral oxygen saturation via pulse oximetry was 99% on room air. A chest x-ray revealed no features of pulmonary edema while an electrocardiogram (ECG) revealed a sinus rhythm with ST elevation in the anteroseptal leads (V1–4), Q waves in the septal leads (V1–2) and peaked T waves in V2–4 indicating an acute anteroseptal ST-Elevation Myocardial Infarction (STEMI) (Fig. 1). A transthoracic echocardiogram showed wall motion abnormalities with interventricular septum and anterior wall hypokinesia. The left ventricle (LV) was dilated with an ejection fraction of 48% (Fig. 2). The right ventricular size was normal whereas the peak pulmonary arterial pressure was elevated at 50 mm/Hg. Lower extremity dopplers revealed no venous thrombus while an assay of cardiac enzymes revealed elevated Troponin I levels of 0.51 ng/ml (normal ≤0.04 ng/ml) and Creatinine Kinase -MB (CK-MB) levels of 54 IU/l (reference range 5–25 IU/l).

Fetal assessment ultrasound done on day 2 of admission revealed an abnormal cerebroplacental ratio (CPR) of 0.57 necessitating delivery by caesarean section. The infant weighed 1 .5kg with Apgar score of 9 at 1 min and 10 at 5 min. Left cardiac catheterization was subsequently done on day 3, approximately 18 h after the last dose of LMWH and showed left ventricular apical ballooning with no evidence of atherosclerosis in the coronaries.

**Fig. 3:**
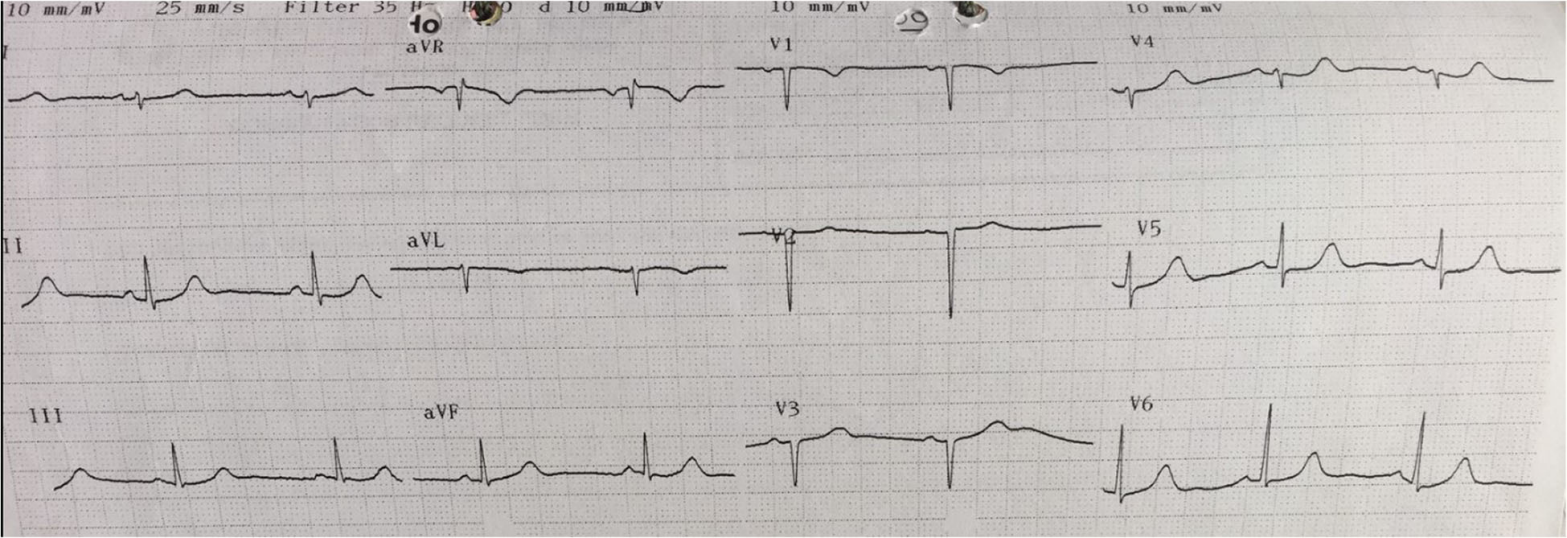
ECG trace showing frontal QRS complex of + 100 degrees. She had QS complexes from V1 to V3 consistent with prior antero-septal myocardial ischemia

**Fig. 4:**
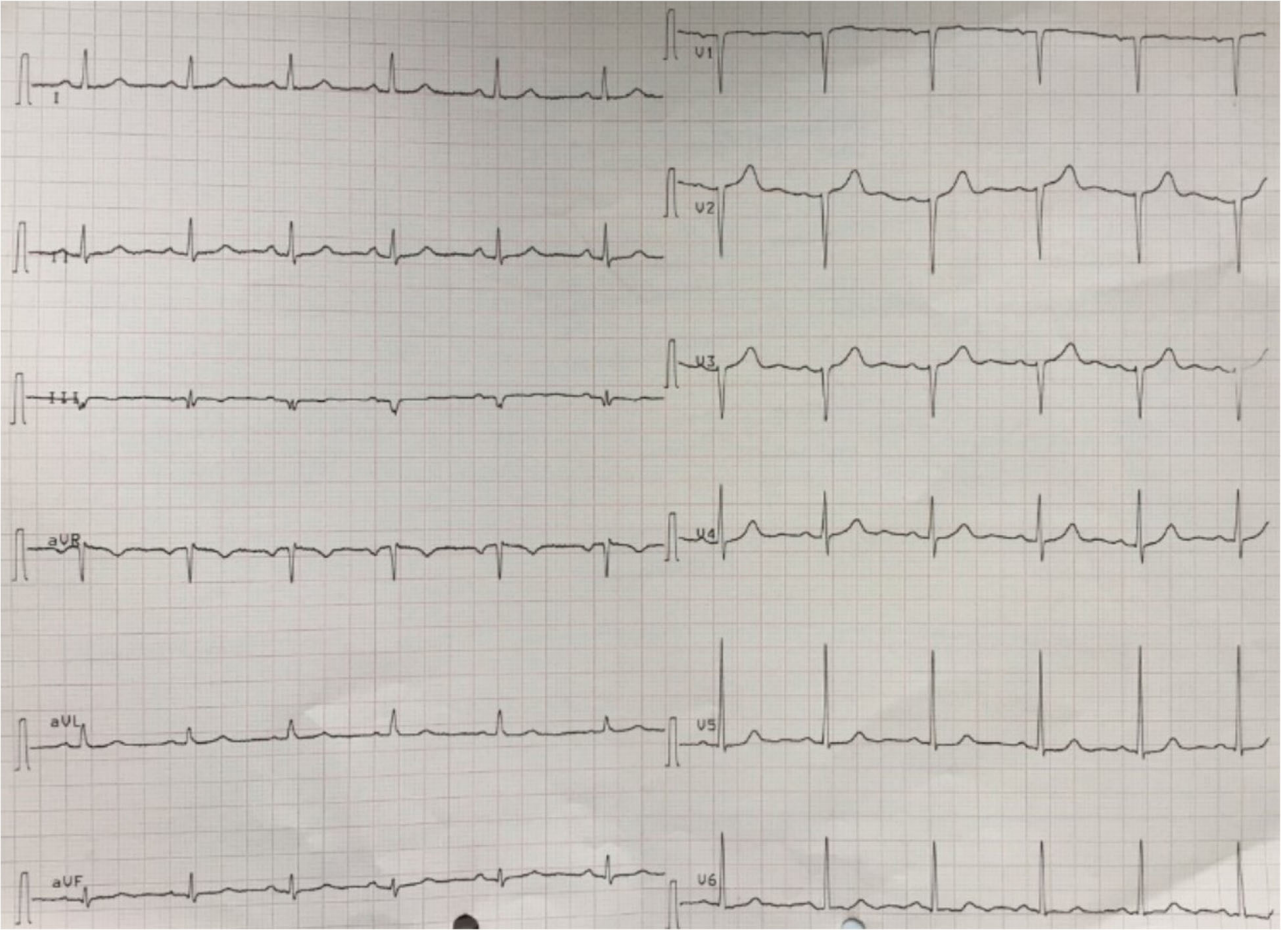
Normal ECG trace following recovery from cardiomyopathy

The LMWH thromboprophylaxis (0 .5mg/kg once daily) was restarted 12 h post-delivery for one week. The blood pressure was controlled with amlodipine (10 mg once daily). Postpartum, she remained normotensive and anti-hypertensive medication was stopped after 6 weeks. Cardiac injury biomarkers normalized by the 10th postpartum day. The ECG done at 6 weeks postpartum showed frontal QRS complex of + 100 degrees with QS complexes from V1 to V3 consistent with recent antero-septal myocardial infarction (Fig. 3) while that done 6 weeks later showed no significant abnormalities (Fig. 4). The echocardiogram at 3 months post-delivery revealed normal cardiac chambers with no regional wall motion abnormalities with a left ventricle ejection fraction of 65%.

## Discussion

Takotsubo cardiomyopathy (TCM) is a rare life-threatening illness that can affect pregnant women. It should be considered a cardiac emergency as it mimics myocardial infarction, peripartum cardiomyopathy, acute myocarditis, and dilated cardiomyopathy and is a diagnosis of exclusion. Takotsubo cardiomyopathy (TCM) typically presents with acute retrosternal chest pain, palpitations and diaphoresis [5]. Affected patients may also have symptoms of left heart failure such as paroxysmal nocturnal dyspnea, orthopnea and dyspnea [1].

The common triggers of TCM include psychosocial stressors with greater occurrence in those with premorbid psychiatric illnesses [6, 13]. Our patient had a history of depressive illness due to a previous adverse pregnancy outcome which may have predisposed her to TCM. In addition, it is possible that the severe preeclampsia caused an acute elevation of blood pressure from the baseline triggering TCM. Increased afterload from hypertension may cause a demand-supply mismatch leading to a type 2 non- ST-elevation myocardial infarction (NSTEMI) [14].

Diagnostic evaluation of TCM includes an electrocardiogram (ECG), cardiac biomarkers, echocardiography and left heart angiography. The ECG abnormalities commonly encountered include ST segment elevation in the anterior and precordial leads with some having ST segment depression [15, 16]. A few patients may have QT interval prolongation, T wave inversion, abnormal Q waves and non-specific abnormalities [15]. The majority of patients will have elevated cardiac troponin levels with normal to mildly elevated creatinine kinase. The natriuretic peptides (brain natriuretic peptide (BNP) and pro-BNP) may be elevated in up to 83% of cases indicating ventricular strain, though to a lesser extent than that seen in acute myocarditis [16].

The typical findings on transthoracic echocardiography include a large area of regional wall motion akinesia of the LV extending beyond the territory of a single coronary artery [17]. There is apical ballooning of the LV with normal basal contractility. The LV ejection fraction is often reduced ranging from 20 to 49% [18]. In addition, there may be mitral regurgitation with or without a systolic anterior motion of the anterior leaflet [17].

The diagnosis of TCM is based on the Mayo clinic diagnostic criteria [1]. This includes presence of transient akinesis, hypokinesis or dyskinesis of the left ventricle with or without apical involvement; this regional ventricular wall motion abnormality extends beyond a single epicardial vascular perfusion territory. Further there is absence of obstructive coronary disease or angiographic evidence of acute plaque rupture. The patient may also have ECG abnormalities (such as ST-segment elevation with or without T-wave inversion) or moderately elevated cardiac troponin levels. Lastly, myocarditis and phaechromocytoma need to be excluded [1, 16]. Our patient had transient ECG abnormalities and no angiographic evidence of acute plaque rupture or vessel occlusion fulfilling the Mayo criteria for TCM.

Up to 25% of TCM cases may develop Left Ventricular Outflow Tract (LVOT) obstruction due to the increased contractility of the base of the heart [18, 19]. Prompt diagnosis by use of echocardiography and timely management results in outcomes comparable to those without LVOT [20]. The detection of LVOT obstruction is important as the patients usually present with hypotension and the use of ionotropic agents may increase the intraventricular pressure gradient and induce cardiogenic shock [18].

Most patients recover normal cardiac function within 4 to 8 weeks [5, 16]. Our patient had resolution of heart failure symptoms with full recovery by 12 weeks post-delivery. Care of these patients is directed at managing their symptoms and medical cardiac optimization with diuretics, angiotensin-converting enzyme inhibitors and beta blockers. Due to association with fetal growth restriction, if on beta blockers, there is need to schedule ultrasounds for fetal growth every 4 weeks.

The resolution of physical or emotional stress usually results in rapid resolution of symptoms though some patients may develop acute complications such as acute heart failure and cardiogenic shock requiring coronary cardiac unit admission and need for invasive techniques such as intra-aortic balloon pump and cardiopulmonary support [10].

The decision on the timing and mode of delivery should be guided by obstetrical reasons. It should involve a multidisciplinary team of cardiologists, obstetricians, neonatologists and psychologists.

Recurrence of TCM in premenopausal women is rare [21]. Close follow up upon discharge is required as some patients may deteriorate and develop major adverse cardiac and cerebrovascular events [1].

In conclusion, TCM is a rare cardiac condition especially in pregnancy. Management challenges for ongoing pregnancies are crowded by safety concerns of the drugs used for managing acute myocardial infarction. Longitudinal studies are needed to evaluate the reproductive history of women with TCM and further assess its association with preeclampsia. However, we strongly advocate for a multidisciplinary approach in order to optimize outcomes for the mother and child.

## Abbreviations

ECG: Electrocardiogram
LMWH: Low Molecular Weight Heparin
LV: Left ventricle
LVOT: Left Ventricular Outflow Tract
TCM: Takotsubo cardiomyopathy

## References

[1] Templin C, Ghadri JR, Diekmann J, Napp LC, Bataiosu DR, Jaguszewski M (2015). Clinical features and outcomes of Takotsubo (stress) cardiomyopathy. N Engl J Med.

[2] Minatoguchi M, Itakura A, Takagi E, Nishibayashi M, Kikuchi M, Ishihara O (2014). Takotsubo cardiomyopathy after cesarean: a case report and published work review of pregnancy-related cases. J Obstet Gynaecol Res.

[3] Salmoirago-Blotcher E, Dunsiger S, Swales HH, Aurigemma GP, Ockene I, Rosman L (2016). Reproductive history of women with Takotsubo cardiomyopathy. Am J Cardiol.

[4] Pelliccia F, Kaski JC, Crea F, Camici PG (2017). Pathophysiology of Takotsubo syndrome. Circulation..

[5] Yaqub Y, Jenkins LA, Nugent KM, Chokesuwattanaskul W (2009). Postpartum depression and apical ballooning syndrome (takotsubo syndrome). J Obstet Gynaecol Can.

[6] Nayeri A, Rafla-Yuan E, Farber-Eger E, Blair M, Ziaeian B, Cadeiras M, et al. Pre-existing psychiatric illness is associated with increased risk of recurrent Takotsubo cardiomyopathy. Psychosomatics. 2017. 10.1016/j.psym.2017.04.008 PMC7658955 28602445

[7] Corrigan FE, Kimmel MC, Jayaram G (2011). Four cases of takotsubo cardiomyopathy linked with exacerbations of psychiatric illness. Innov Clin Neurosci.

[8] Virani SS, Khan AN, Mendoza CE, Ferreira AC, de Marchena E (2007). Takotsubo cardiomyopathy, or broken-heart syndrome. Tex Heart Inst J.

[9] Ruiz S, Martinez-Marin M, Luque P, Nassar N, Oros D (2017). Takotsubo cardiomyopathy after cesarean section: a case report and literature review. J Obstet Gynaecol Res.

[10] Suzuki T, Nemoto C, Ikegami Y, Yokokawa T, Tsukada Y, Abe Y (2014). Development of takotsubo cardiomyopathy with severe pulmonary edema before a cesarean section. J Anesth.

[11] Smith JM, Lowe RF, Fullerton J, Currie SM, Harris L, Felker-Kantor E (2013). An integrative review of the side effects related to the use of magnesium sulfate for pre-eclampsia and eclampsia management. BMC Pregnancy Childbirth..

[12] Chollat C, Le Doussal L, de la Villeon G, Provost D, Marret S (2017). Antenatal magnesium sulphate administration for fetal neuroprotection: a French national survey. BMC Pregnancy Childbirth.

[13] Zdanowicz JA, Utz AC, Bernasconi I, Geier S, Corti R, Beinder E (2011). "broken heart" after cesarean delivery. Case report and review of literature. Arch Gynecol Obstet.

[14] Picariello C, Lazzeri C, Attana P, Chiostri M, Gensini GF, Valente S (2011). The impact of hypertension on patients with acute coronary syndromes. Int J Hypertens.

[15] Otani Y, Tokunaga K, Kawauchi S, Inoue S, Watanabe K, Kiriyama H (2016). Cerebral infarction arising from Takotsubo cardiomyopathy: case report and literature review. NMC Case Rep J.

[16] Rozema T, Klein LR (2016). Takotsubo cardiomyopathy: a case report and literature review. Cardiol Young.

[17] Gupta S, Gupta MM (2018). Takotsubo syndrome. Indian Heart J.

[18] Roshanzamir S, Showkathali R (2013). Takotsubo cardiomyopathy a short review. Curr Cardiol Rev.

[19] Yalta K, Yilmaztepe M, Zorkun C (2018). Left ventricular dysfunction in the setting of Takotsubo cardiomyopathy: a review of clinical patterns and practical implications. Card Fail Rev.

[20] De Backer O, Debonnaire P, Gevaert S, Missault L, Gheeraert P, Muyldermans L (2014). Prevalence, associated factors and management implications of left ventricular outflow tract obstruction in takotsubo cardiomyopathy: a two-year, two-center experience. BMC Cardiovasc Disord.

[21] Hefner J, Csef H, Frantz S, Glatter N, Warrings B (2015). Recurrent Tako-Tsubo cardiomyopathy (TCM) in a pre-menopausal woman: late sequelae of a traumatic event?. BMC Cardiovasc Disord.

